# High frequency of Lamivudine and Telbivudine resistance mutations in hepatitis B virus isolates from human immunodeficiency virus co-infected patients on highly active antiretroviral therapy in Bucaramanga, Colombia

**DOI:** 10.3389/fmicb.2023.1202342

**Published:** 2023-07-24

**Authors:** Henry Bautista-Amorocho, Jorge Alexander Silva-Sayago, Jirehl Picón-Villamizar

**Affiliations:** Grupo de Investigación en Manejo Clínico CliniUDES, Facultad de Ciencias de la Salud, Universidad de Santander, Bucaramanga, Colombia

**Keywords:** HIV, HBV, coinfection, NRTI, antiviral, resistance, mutation

## Abstract

Hepatitis B virus (HBV) antiviral Resistance-Associated Mutations (RAMs) in human immunodeficiency virus (HIV) coinfected patients undergoing highly active antiretroviral therapy (HAART) are complex and incompletely understood. We aimed to determine the prevalence of HBV coinfection, HBV genotypes, and RAMs in a cohort of people living with HIV (PLWH) in the northeastern region of Colombia. This cross-sectional study was carried out between February 2013 and February 2014. Virological, immunological and HAART data were collected from clinical records. In-house nested PCR and Sanger sequencing of the HBV *pol* gene were used to identify coinfections, genotypes, RAMs and HBV s antigen (HBsAg) escape mutants. Among 275 PLWH, HBV coinfection was confirmed in 32 patients (11.6%), of whom nine (28.2%) were HBsAg positive (active hepatitis B), and 23 (71.8%) were occult hepatitis B infections (OBI). All HBV sequences (n = 23) belonged to the genotype F3. Among HIV/HBV coinfections, 71.9% had CD4+ T cell counts above 200 cells/mm^3^ and 37.5% had undetectable HIV viral loads. The RAMs rtL80I, rtL180M, and rtM204V, which confer resistance to Lamivudine/Telbivudine and partially resistant to Entecavir, were found in all HBV isolates. An unknown rt236Y mutation to Tenofovir was also identified. Most patients under HAART received first-generation HBV antiviral therapy with a low genetic barrier to resistance. Antiviral Drug-associated Potential Vaccine-escape Mutations (ADAPVEMs) in the *S* gene were observed in all isolates ranging from 1–20 amino acid substitutions. However, no vaccine escape mutants were detected. In Conclusion, these findings highlight the importance of HBV molecular screening, antiviral resistance monitoring and new guidelines for PLWH to overcome RAMs and prevent HBV-related liver disease.

## 1. Introduction

Human immunodeficiency virus (HIV) infection remains a major global public health problem. According to UNAIDS there were 38.4 million (33.9 million–43.8 million) people living with HIV (PLWH) in 2021 and 40.1 million (33.6 million–48.6 million) have died from acquired immunodeficiency syndrome (AIDS) related illnesses since the beginning of the epidemic (UNAIDS, 2023). Despite global efforts to prevent transmission, there is still no effective curative intervention or an effective vaccine to control the spread of the virus. The therapeutic approach against HIV focuses on inhibiting viral replication in CD4 + T cells through the use of highly active antiretroviral therapy (HAART) to allow partial recovery of the immune system and strengthen its ability to fight off opportunistic infections (Vittinghoff et al., 1999). HAART is a combination of three or more antiretroviral drugs and includes more than 30 molecules divided into six groups, according to their mechanism of action on HIV replication (Saag et al., 2018).

Widespread use of HAART in many countries has led to substantial reduction in AIDS-related deaths and increased life expectancy (Mocroft et al., 2003; Tanser et al., 2013). However, chronic hepatotropic virus infection is a major cause of morbidity and mortality in PLWH (Palella et al., 1998; Vallet-Pichard and Pol, 2004). Hepatitis B virus (HBV) is generally transmitted by percutaneous or mucosal exposure to infected blood and other body fluids. The mechanisms of transmission are the same as those for HIV, but HBV is 50–100 times more infectious (Thio et al., 2002). The World Health Organization (WHO) estimates the occurrence of 1.5 million new cases of HBV per year. In 2019, 296 million people were chronically infected and there were more than 820,000 HBV-related deaths worldwide (Platt et al., 2020; WHO, 2021). In addition, HIV/HBV co-infection is common in endemic areas with approximately 5–15% of PLWH chronically infected with hepatitis B (CHB) (Martín-Carbonero et al., 2011). CHB adversely affects the natural history of HIV and accelerates the progression to end-stage liver disease, including hepatic decompensation, cirrhosis, and hepatocellular carcinoma (HCC) (Martín-Carbonero et al., 2011).

Dual antiviral therapy is recommended for the treatment of HIV/HBV co-infection. HAART can therefore include two nucleoside/nucleotide reverse transcriptase inhibitors (NRTI) with anti-HBV activity, such as Tenofovir Disoproxil Fumarate (TDF), Tenofovir Alafenamide (TAF), Lamivudine (LAM), or Emtricitabine (FTC) (Shire and Sherman, 2005; Lampertico et al., 2017). However, inadequate suppression of viral replication in patients undergoing pharmacological treatment leads to in the development of NRTI-resistant mutations within the RT domain of the HBV *pol* gene (Archampong et al., 2017). Thus, antiviral therapy should include a correct diagnosis of HBV infection and antiviral susceptibility profile to prevent the emergence of drug-resistant mutations and resumption of active viral replication (He et al., 2015).

In Colombia, the number of PLWH has increased dramatically from 35,000 in 2012 to 134,636 in 2021, with a national prevalence of 0.3%. Despite public health policies and active screening programs in at-risk populations, approximately 30% of cases are underdiagnosed or unreported. In addition, the prevalence of HIV/HBV coinfection in the country was 4.77% of which 1.89% were CHB cases (CAC, 2021). However, coinfection increased threefold when the nucleic acid amplification testing was used, regardless of the HBV serologic profile, identifying 8.7% of cases as occult hepatitis B virus infection (OBI) (Bautista-Amorocho et al., 2014). OBI refers to the presence of HBV DNA in liver tissue or blood in the absence of detectable hepatitis B surface antigen (HBsAg) (Raimondo et al., 2019). Given the low sensitivity of HBsAg screening among PLWH, it is likely that a large proportion of HBV infections in this group remain undetected. In addition, the first-line HAART regimens often includes LAM in HIV mono-infected individuals, who are at increased risk for HBV NRTI-resistant mutations. In Colombia and Latin America, there is no evidence on the prevalence and genetic profile of HBV RAMs in co-infected PLWH. Therefore, we aimed to determine the prevalence of coinfection and the antiviral resistance patterns in the RT region of the HBV *pol* gene in a cohort of PLWH on HAART in the city of Bucaramanga, province of Santander.

## 2. Materials and methods

### 2.1. Study design and research subjects

We conducted a descriptive cross-sectional study between February 2013 and February 2014 in the city of Bucaramanga, province of Santander, located in the northeastern region of Colombia. Samples were collected using convenience sampling and included PLWH diagnosed at the IPS Medical Centre in the city. Blood (5 mL) was collected from each participant, along with demographic, clinical and laboratory parameters, including HBV serologic markers determined by enzyme-linked immunosorbent assay, HIV viral load measured with the Real Time HIV-1 Amplification Reagent Kit (Abbott, IL, USA) with a detection range of 50 to 10,000,000 copies/mL, CD4+ T-cell counts, and HAARTcombinations.

Inclusion criteria were as follows: HIV antibody positive confirmed by ELISA and Western Blot, duration of HIV infection over 6 months, and access to patient medical records. Exclusion criteria were as follows: pregnant and breastfeeding women, mental illness and patients under 18 years of age.

### 2.2. HIV and hepatitis B virus coinfection definitions

Active HBV infection was defined as positive of HBsAg serologic marker by enzyme immunoassay or rapid diagnostic tests in PLWH with or without viral DNA amplification by nested PCR. OBI was confirmed when HBV DNA was amplified by nested PCR in the absence of HBsAg and independent of other HBV serologic markers.

### 2.3. Nested PCR for hepatitis B virus identification and sequencing

Total DNA was purified from 1 mL of serum using a QIAamp Ultra Sense Virus Kit (QIAgen, MD, USA) according to manufacturer’s instructions. Nucleic acid purity and concentration were determined by absorbance measurements 260/280 nm using a NanoDrop 2000C (Thermo Fisher, CA, USA). DNA aliquots were stored at −80°C until PCR processing.

Detection of HBV co-infection in HIV serum samples was carried out using an in-house nested PCR to amplify a 1,027 bp fragment of the *pol* gene containing the entire reverse transcriptase (RT) region using primers as described in Table 1. The first round of amplification was performed in a total volume of 25 μL, containing 0.2 μM of each primer, 11.5 μL of sample DNA, and 12.5 μL of enzyme OneTaq Hot Start 2X MM Polymerase (New England Biolabs, MA, USA) according to the manufacturer’s instructions. Cycling conditions were 94°C for 30 s, 35 cycles of 94°C for 15 s, 60°C for 15 s, and 68°C for 2 min, ending with 68°C for 10 min. Amplification was carried out using the ProFlex PCR System (Applied Biosystems, Thermo Fisher, CA, USA). The second round of PCR was performed under the same conditions, except that 1 μL of the first-round product was added to the reaction tubes. PCR products were separated by agarose gel electrophoresis and detected using SYBR Safe DNA Gel Stain and a 1 kb GeneRuler DNA Ladder (Thermo Scientific, CA, USA). Positive samples were confirmed by a second assay using the same conditions as above. Fragments were purified using the QIAquick PCR Purification Kit (QIAgen, MD, USA) and sequenced bidirectionally using the inner primers described in Table 1.

**Table 1:** Inner and outer primers for HBV RT domain nested PCR and sequencing.

Primer name	Primer sequence 5′-3′	HBV genome position (from *Eco*RI site)	Fragment length (bp)
HBV RT Outer F1	CTGCTGGTGGCTCCAGTTC	55–75	1,876
HBV RT Outer R1	GCTCCAAATTCTTTATAAGGGTCAATG	1,930–1,903	
HBV RT Inner F2	CATCAGGACTCCTAGGACCCCT	168–189	1,027
HBV RT Inner R2	TGCGTCAGCAAACACTTGGCA	1,194–1,174	

### 2.4. Hepatitis B virus genotyping

PCR fragments corresponding to the RT domain of the *pol* gene were sequenced using the Sanger platform 3730XL (Applied Biosystems, Thermo Fisher, CA, USA). Raw HBV DNA sequences from co-infected PLWH and two HBsAg (+) blood donors without prior NRTI treatment were automatically assembled using SeqMan Ultra (DNA Lasergene v17.1). The trimmed sequences were aligned to the consensus derived from representative HBV reference sequences from NCBI using Clustal W software. Local HBV isolates were deposited in the NCBI GenBank under the accession numbers: OQ262971-OQ262995.

HBV genotypes and subgenotypes were determined by phylogenetic inference using Molecular Evolutionary Genetics Analysis (MEGA, V.11) (Kumar et al., 2018). HBV isolates and sequences of reference genotypes and subgenotypes retrieved from GenBank were used for the analysis. The selection of the evolutionary model was based on the lower Bayesian Information Criterion (BIC) score, and the phylogenetic tree was generated by the Maximum Likelihood (ML) method, using the Subtree Pruning Regrafting (SPR) heuristic algorithm. The confidence level of the inferred tree was determined by bootstrapping with 1,000 replicates. The resulting tree was exported in Nowick’s format and edited using FigTree (v. 1.4.4).

### 2.5. Identification of antiviral resistance-associated mutations in HBV *pol* gene and antiviral drug-associated potential vaccine-escape mutations in the *S* gene

HBV resistance and compensatory mutations to NRTIs were identified using the Geno2pheno [HBV] 2.0, drug resistance tool developed by the Max Planck Institute.^1^ HBV sequences from each co-infected individual were included in data collection. Mutations were assessed at 11 different positions (rtL80, rtI169, rtV173, rtL180, rtA181, rtS184, rtA194, rtS202, rtM204, rtN236, and rtM250), starting at codon 344 within the open reading frame (ORF) of the *pol* gene. Amino acid substitution profiles associated with INTR resistance were described according to the Clinical Practice Guidelines for the management of HBV infection (Lampertico et al., 2017). The ORF of the HBV envelope (*S*) gene was also analyzed to identify escape mutations in the “a determinant” within the Major Hydrophilic Region (MHR) and ADAPVEMs. Similarly, the deduced amino acid sequences for the *P* and *S* genes were aligned with the consensus sequences obtained from several annotations of the F genotype to exclude genotype-related polymorphisms and variations.

### 2.6. Statistical analysis

Demographic, clinical, and RAMs data were analyzed for all study participants. Continuous variables were presented as range, median and interquartile range for CD4+ cell counts and HIV viral loads, while categorical variables were presented as percentages. Comparisons between mono-infected and co-infected groups for categorical and continuous variables were assessed using Chi-square and Mann–Whitney tests, respectively. Statistical significance was set at value of *p* ≤ 0.05. Analyses were performed in Python using the pandas and SciPy Stats libraries.

### 2.7. Ethical considerations

The study was approved by the Ethics Committee of the Universidad de Santander (approval 015-2012), and written informed consent was obtained from all the participants before sampling and data collection. All procedures performed during the study were in accordance with the Declaration of Helsinki and the Colombian regulations on ethics in clinical research (Resolution 8430 1993).

## 3. Results

### 3.1. Characteristics of the HIV/HBV co-infections and HIV mono-infections in the study population

A total of 275 PLWH were enrolled in the study, with a median age of 38 years (30–44) and 65.1% male. All HIV transmissions were sexually acquired. The majority of the participants were heterosexual (66.2%), followed by homosexual (15.6%) and bisexual (5.8%). HIV infection was classified according to the Centers for Disease Control and Prevention, with 32.4% of patients in C category (AIDS state), 34.5% in category B (symptomatic), and 30.2% in category A (asymptomatic). CD4+ T-cell counts showed that 24.7% of patients had less than 200 cells/mm^3^, 46.6% had between 200–500 cells/mm^3^, and 28.7% had more than 500 cells/mm^3^. Plasma HIV viral load was classified as undetectable (<50 copies/mL) in 43.2% of patients, low (51–10,000 copies/mL) in 34.2% and high (>100,000 copies/mL) in 7.3%. HBV co-infection was confirmed in 32 HIV samples (prevalence 11.6%), of which nine (28.2%) were HBsAg positive (active hepatitis B) and 23 (71.8%) were OBI. Among HIV/HBV co-infected patients, HBV serologic markers showed that 28.1% were HBsAg positive and 40.6% were total anti core antibodies (anti-HBc) positive. Surprisingly, 31.3% had antibodies against HBV S antigen (anti-HBs) and 9.4% were hepatitis B vaccinated. No significant differences were observed between HIV/HBV co-infected and HBV mono-infected patients, except for the positivity of the serological markers anti-HBc and HBsAg (*p* ≤ 0.05) (Table 2).

**Table 2:** Demographic and clinical characteristics of participating subjects (*n* = 275).

Variable	Type of patient			Value of *p*
	Total	Co-infected HIV/HBV	HIV positive	
	*n* = 275 (100%)	*n* = 32 (11.6%)	*n* = 243 (88.4%)	
Gender
Male	179 (65.1%)	21 (65.6%)	158 (65%)	1.0^a^
Age (median years-IQR)				
	38 (30–44)	37 (31–42)	38 (29–44)	0.73^b^
Risk factor
Heterosexual	182 (66.2%)	22 (68.8%)	160 (65.8%)	0.52^a^
Homosexual	24 (15.6%)	8 (25%)	35 (14.4%)	
Bisexual	8 (5.8%)	2 (6.2%)	14 (5.8%)	
ND	31 (12.4%)	0 (0%)	34 (14.0%)	
CDC HIV classification
A	83 (30.2%)	10 (31.3%)	73 (30.0%)	0.62^a^
B	95 (34.5%)	13 (40.6%)	82 (33.7%)	
C	89 (32.4%)	9 (28.1%)	80 (32.9%)	
ND	8 (2.9%)	0 (0%)	8 (3.3%)	
CD4^+^ count (cells/mm^3^)
Median - IQR	342 (200–532)	316 (183–447)	347 (203–532)	
<200	68 (24.7%)	9 (28.1%)	59 (24.3%)	0.70^a^
200–500	128 (46.6%)	16 (50%)	112 (46.1%)	
>500	79 (28.7%)	7 (21.9%)	72 (29.6%)	
HIV viral load (copies/mL)
Median-IQR	84 (40–8,615)	702.5 (40–32188.8)	80 (40–7220.5)	
Undetectable ≤50	119 (43.2%)	12 (37.5%)	107 (44.0%)	0.20^a^
Low 51–10,000	94 (34.2%)	9 (28.1%)	85 (35.0%)	
Mid 10,001-99,999	42 (15.3%)	9 (28.1%)	33 (13.6%)	
High >100,000	20 (7.3%)	2 (6.3%)	18 (7.4%)	
HBV serologic markers
HBsAg				
Negative	266 (96.7%)	23 (71.9%)	243 (100%)	<0.05^a,*^
Positive	9 (3.3%)	9 (28.1%)	0 (0%)	
Anti-HBc				
Negative	206 (74.9%)	19 (59.4%)	187 (77%)	0.05^a,*^
Positive	69 (25.1%)	13 (40.6%)	56 (23%)	
Anti-HBs				
Negative	168 (61.1%)	22 (68.7%)	146 (60.1%)	0.45^a^
Positive	107 (38.9%)	10 (31.3%)	97 (39.9%)	
HBV vaccination				
No	54 (19.6%)	8 (25%)	46 (18.9%)	0.29^a^
Yes	47 (17.1%)	3 (9.4%)	44 (18.1%)	
ND	174 (63.3%)	21 (65.6%)	153 (63.0%)	

ND, No data; HBsAg, hepatitis B surface antigen; Anti-HBc, hepatitis B core antibody; Anti-HBs, hepatitis B surface antibody; CDC, Center for Disease Control and Prevention; IQR, interquartile range. ^a^Chi square test; ^b^Mann–Whitney test. *Statistical differences were observed.

### 3.2. Highly active antiretroviral therapy in PLWH

At baseline, 89.4% (*n* = 246) of PLWH were receiving HAART and most combinations (99.6%) included an NRTI. In 156/246 (56.7%) regimens contained NRTI+NNRTI (non-nucleos(t)ides reverse transcriptase inhibitors), of which 62.2% received LAM, Zidovudine (ZDV), and Efavirenz (EFV). The NRTI + PI (protease inhibitors) family of drugs were administered in 56/246 individuals (22.7%). Of these, LAM, ZDV, Lopinavir (LPV), and Ritonavir (RTV) were the most commonly used in 42.9% of patients. The results also highlighted 23 patients receiving more than two molecules from the same family of NRTIs and ten subjects treated with drugs from three different HAART regimens, such as NRTI+NNRTI+PI. Only one participant benefited from the integrase inhibitor Raltegravir (RAL) with the protease inhibitor LPV. In summary, the data show that 38 different HAART regimens are being used in PLWH from Colombia (Table 3).

**Table 3:** Frequency of HAART in PLWH.

HAART	*n*	%
NRTIs
LAM + ZDV	18	6.5
4dT + ddl	2	0.7
LAM + ABC	1	0.4
LAM + ZDV + ABC	1	0.4
ZDV + ddl	1	0.4
Subtotal	23	9.3
NRTIs + NNRTIs		
LAM + ZDV + EFV	97	39.4
LAM + ABC + EFV	23	9.3
LAM + ZDV + NVP	20	8.1
LAM + EFV	7	2.8
LAM + 4dT + EFV	4	1.6
LAM + ddl + EFV	1	0.4
LAM + ABC + NVP	1	0.4
LAM + 4dT + NVP	1	0.4
ZDV + ABC + EFV	1	0.4
ABC + ddl + EFV	1	0.4
Subtotal	156	63.4
NRTI + PI		
LAM + ZDV + LPV + RTV	24	9.8
ABC + ddl + LPV + RTV	6	2.4
LAM + ZDV + NFV	5	2.0
LAM + ABC + LPV + RTV	3	1.2
LAM + 4dT + NFV	3	1.2
LAM + 4dT + LPV + RTV	3	1.2
ABC + LPV + RTV	2	0.8
LAM + LPV + RTV	2	0.8
LAM + ZDV + RTV	1	0.4
LAM + ABC + RTV	1	0.4
LAM + NFV	1	0.4
ZDV + LPV	1	0.4
ZDV + ddl + LPV + RTV	1	0.4
ZDV + LPV + RTV	1	0.4
ABC + 4dT + LPV + RTV	1	0.4
4dT + LPV + RTV	1	0.4
Subtotal	56	22.8
NRTI + NNRTI + PI		
LAM + ZDV + ATZ	4	1.6
ABC + ddl + RTV + FSM	2	0.8
LAM + ABC + ATZ	1	0.4
LAM + ZDV + NFV	1	0.4
LAM + ZDV + NVP + NFV	1	0.4
LAM + ZDV + NVP + RTV + FSM	1	0.4
Subtotal	10	4.1
PI + II		
LPV + RAL	1	0.4
Subtotal	1	0.4
Total	246	100

HAART, highly active antiretroviral therapy; II, Integrase inhibitors; NRTI, Nucleos (t)ide reverse transcriptase inhibitors; NNRTI, Non nucleos(t)ides reverse transcriptase inhibitors; PI, Protease inhibitors; ABC, Abacavir; ATZ, Atazanavir; ddl, Didanosine; EFV, Efavirenz; FSM, Fosamprenavir; LAM, Lamivudine; LPV, Lopinavir; NVP, Nevirapine; NFV, Nelfinavir; RAL, Raltegravir; RTV, Ritonavir; ZDV, Zidovudine; 4dT, Stavudine.

### 3.3. HBV genotyping analysis

**Figure 1:**
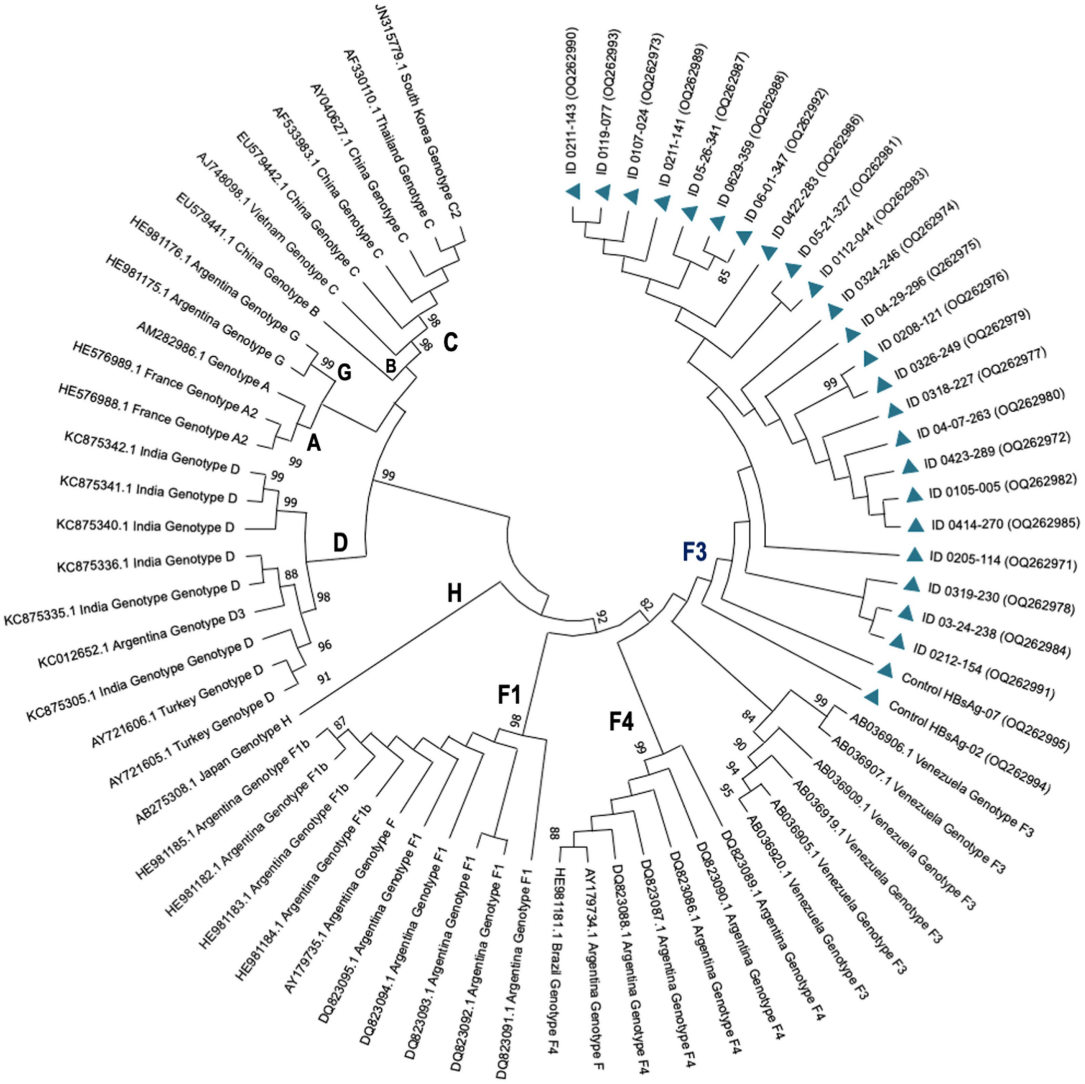
Phylogenetic tree showing HBV genotypes and subgenotypes by maximum likelihood inference. HBV isolates from PLWH in Colombia are represented by codes OQ262971-OQ262995 (blue triangles). Reference strains are represented by their accession numbers as they appear in GenBank followed by the country of isolation and their corresponding genotypes. Probability values ≥0.80 are indicated at the nodes of the tree.

A total of 24 HIV samples tested positive for HBV using nested PCR, with only one sample showing positive results for HBsAg (patient ID 0119-077). High quality DNA was successfully extracted from 23 samples, allowing the collection of DNA data based on electropherogram profiles for subsequent phylogenetic analyses. Based on evolutionary clustering, all patients were infected with the HBV genotype F3, with clade support after 1,000 bootstrap replicates of 98 and 75%, respectively (Figure 1).

### 3.4. Antiviral resistance-associated mutations and ADAPVEMs

**Figure 2:**
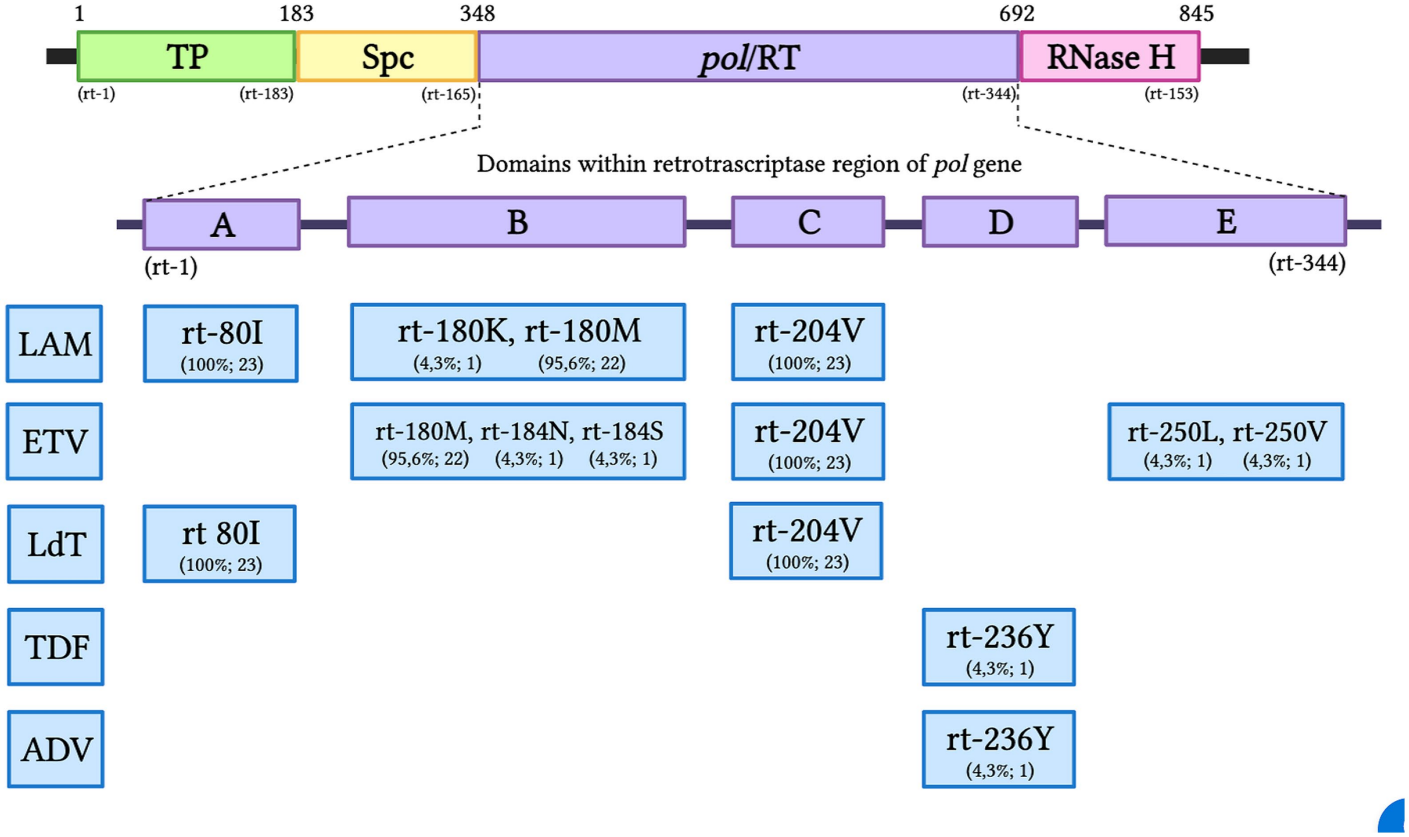
The HBV *pol* gene with the RT region and the A-E domains of Antiviral Resistance-Associated Mutations (RAMs). The resistance pattern found for each NRTI is presented as a percentage and the total number of subjects in the Colombian HIV/HBV cohort: TP, Terminal Profile; SPC, Space region; Pol/RT, Polymerase/Reverse Transcriptase; LAM, Lamivudine; ETV, Entecavir; LdT, Telbivudine; TDF, Tenofovir; ADV, Adefovir.

**Figure 3:**
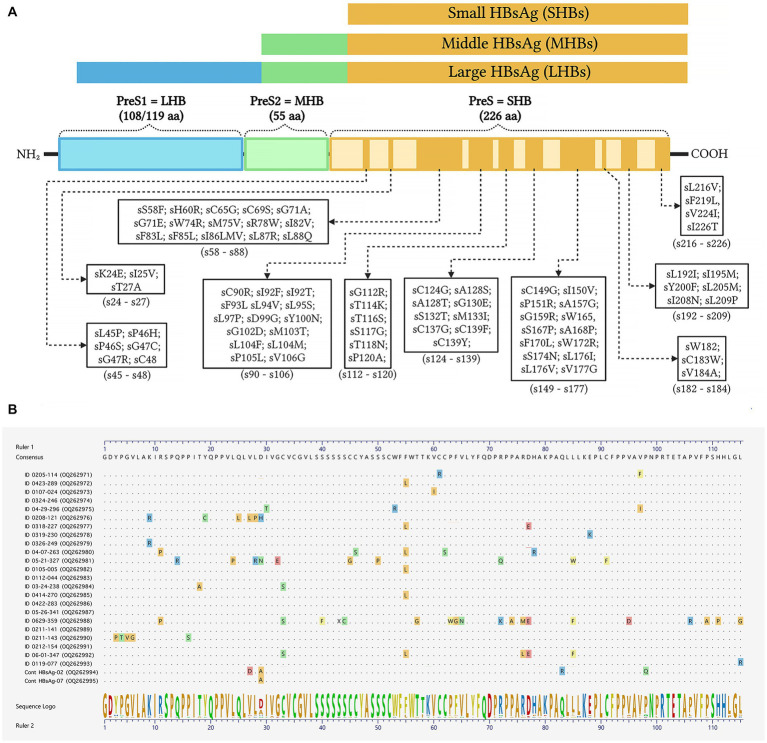
Schematic representation of amino acid substitutions in the HBV envelope/surface proteins. **(A)** Color bars show the large HBV surface antigen (LHBs), middle surface antigen (MHBs), and small surface antigen (SHBs) with antiviral drug-associated potential vaccine-escape mutations (ADAPVEMs). Each ADAPVEM found in the present study is plotted along the SHBs according to the output of the geno2pheno system outputs. **(B)** Clustal W alignments of the SHBs at amino acid positions 21–136. The sequences confirmed all amino acid substitutions found in PLWH co-infected with HBV (GenBank accession numbers OQ262971-OQ262995). The consensus amino acid sequence was derived from the same F genotype strains used to construct the phylogenetic tree in Figure 1.

Inferred amino acid sequences at position 27–344 of the HBV RT domain of the *pol* gene and 21–136 of the envelope (*S*) gene (partially overlapping *pol* gene within the MHR) were used for NRTI and HBsAg mutant scape analyses. All HBV sequences (*n* = 23) showed resistance and/or compensatory mutations that were typically associated with prolonged NRTI treatment. The most common amino acid substitution profiles are shown in Table 4 and are as follows: rt80I, rt180M, rt204V in 91.3% and rt180M, rt204V in 4.3%, both profiles associated with cross-resistance to LAM and Telbivudine (LdT) and partial resistance to Entecavir (ETV) (Figure 2). In addition, rt236Y was detected as an unknown mutation in TDF. ADAPVEMs in the envelope (*S*) gene were observed in all isolates ranging from 1–20 amino acid substitutions (Figure 3). However, no vaccine escape mutants were detected.

**Table 4:** NRTI resistance and/or compensatory mutations in the RT domain and ADAPVEMs in HBV isolates of PLWH in the Colombian cohort.

Patient ID	HIV viral load (copies/mL)	CD4+ counts (cells/mL)	HAART regimen	NRTI resistant mutation patterns in the RT domain of *pol* gene in HBV					Mutations in the envelope (*S*) gene of HBV
				Lamivudine	Adefovir	Entecavir	Tenofovir	Telbivudine	ADAPVEMs
0205–114	448	1,419	LAM + ZDV + EFV	(rt180M, rt204V, rt80I)	--	Partially resistant (rt204V, rt180M)	--	(rt204V, rt80I)	sG47R, sM75V, sM103T, sC139F, sI195M
0423–289	144	114,733	LAM + ZDV + EFV	(rt180M, rt204V, rt80I)	--	Partially resistant (rt204V, rt180M)	--	(rt204V, rt80I)	sL97P, sW182*, sI195M
0107–024	659	40	LAM + ABC + EFV	(rt180M, rt204V, rt80I)	--	Partially resistant (rt204V, rt180M)	--	(rt204V, rt80I)	sI25V, sG102D, sI195M
0324–246	117	165	LAM + 4dT + NVP	(rt180M, rt204V, rt80I)	--	Partially resistant (rt204V, rt180M)	--	(rt204V, rt80I)	sI195M
04–29-296	201	40	LAM + ZDV + EFV	(rt180M, rt204V, rt80I)	--	Partially resistant (rt204V, rt180M)	--	(rt204V, rt80I)	sF85L, sL95S, sC139Y, sI195M
0208–121	297	37,762	LAM + ZDV + EFV	(rt180M, rt204V, rt80I)	--	Partially resistant (rt204V, rt180M)	--	Resistant (rt204V, rt80I)	sT27A, sG47R, sC69S, sG71A, sR78W, sC90R, sA128T, sS167P, sI195M
0318–227	590	16,312	LAM + ABC + LPV + RTV	(rt180M, rt204V, rt80I)	--	Partially resistant (rt204V, rt180M)	--	(rt204V, rt80I)	sL97P, sP120A, sI150V, sV184A, sI195M
0319–230	220	40	LAM + ZDV + EFV	(rt180M, rt204V, rt80I)	--	Partially resistant (rt204V, rt180M)	--	(rt204V, rt80I)	sI82V, sG130E, sI195M
0326–249	335	40	LAM + ABC + LPV + RTV	(rt180M, rt204V, rt80I)	--	Partially resistant (rt204V, rt180M)	--	(rt204V, rt80I)	sC90R, sS167P, sI195M
04–07-263	294	40	LAM + ZDV + NVP	(rt180K, rt204V, rt80I)	--	Partially resistant (rt204V, rt184N, rt180K)	--	(rt204V, rt80I)	sW74R, sL88Q, sL97P, sL104F, sG112R, sT114K, sS132T, sW165*, sA168P, sW172R, sL176I, sI195M
05–21-327	72	73,327	LAM + ZDV + EFV	(rt180M, rt204V, rt80I)	--	Resistant (rt184S, rt204V, rt180M)	--	(rt204V, rt80I)	sC65G, sG71E, sW74*, sF83L, sL87R, sI92T, sY100N, sC124G, sM133I, sC137G, sF170L, sL176V, sV177G, sI195M, sL216V, sF219L
0105–005	745	40	LAM + ZDV + LPV + RTV	(rt180M, rt204V, rt80I)	--	Partially resistant (rt204V, rt180M)	--	(rt204V, rt80I)	sL97P, sS174N, sI195M
0112–044	217	60,334	LAM + ZDV + EFV	(rt180M, rt204V, rt80I)	Unknown mutation (rt236Y)	Partially resistant (rt204V, rt250L, rt180M)	Unknown mutation (rt236Y)	(rt204V, rt80I)	sL192I, sI195M, sI208N, sL216V, sV224I, sI226T, s*227Y
03–24-238	759	40	LAM + ZDV + EFV	(rt180M, rt204V, rt80I)	--	Partially resistant (rt204V, rt180M)	--	(rt204V, rt80I)	sH60R, sM75K, sI195M, sL209P, sL216V
0414–270	351	30,331	LAM + ZDV + EFV	(rt180M, rt204V, rt80I)	--	Partially resistant (rt204V, rt180M)	--	(rt204V, rt80I)	sL97P, sI195M
0422–283	29	78	LAM + ZDV + EFV	(rt180M, rt204V, rt80I)	--	Partially resistant (rt204V, rt180M)	--	(rt204V, rt80I)	sP46S, sI195M
05–26-341	178	45,108	LAM + 4dT + LPV + RTV	(rt180M, rt204V, rt80I)	--	Partially resistant (rt204V, rt180M)	--	(rt204V, rt80I)	sI195M, sF219L
0629–359	687	40	LAM + ZDV + NFV	(rt180M, rt204V, rt80I)	--	Partially resistant (rt204V, rt180M)	--	(rt204V, rt80I)	sM75K, sI86LMV, sF93L, sL94V, sD99G, sL104M, sP105L, sV106G, sC107*, sT114K, sT116S, sT118N, sP120A, sA128S, sC137*, sC149G, sP151R, sA157G, sI195M, sF219L
0211–141	446	209	LAM + ZDV + NVP	(rt180M, rt204V, rt80I)	--	Partially resistant (rt204V, rt180M)	--	(rt204V, rt80I)	sI195M
0211–143	403	40	LAM + ZDV + EFV	(rt180M, rt204V, rt80I)	--	Partially resistant (rt204V, rt180M)	--	(rt204V, rt80I)	sL45P, sP46H, sG47C, sC48W, sS58F, sI195M
0212–154	34	29,058	LAM + ABC + EFV	(rt180M, rt204V, rt80I)	--	Partially resistant (rt204V, rt180M)	--	(rt204V, rt80I)	sG159R, sC183W, sI195M, sY200F, sL205M, sL209P
06–01-347	153	42,414	LAM + ZDV + ABC	(rt180M, rt204V, rt80I)	--	Partially resistant (rt204V, rt180M)	--	(rt204V, rt80I)	sM75K, sI92F, sL97P, sT114K, sS117G, sP120A, sA128S, I195M, sF219L
0119–077	342	319	LAM + ddl + EFV	(rt180M, rt204V, rt80I)	--	(rt250V, rt204V, rt180M)	--	(rt204V, rt80I)	sK24E, sI195M
Control HBsAg-02			Treatment naïve	--	--	--	--	--	sC69*
Control HBsAg-07			Treatment naïve	--	--	--	--	--	-

ABC, Abacavir; ddl, Didanosine; EFV, Efavirenz; LAM, Lamivudine; LPV, Lopinavir; NVP, Nevirapine; NFV, Nelfinavir; RTV, Ritonavir; ZDV, Zidovudine; 4dT, Stavudine.

## 4. Discussion

In this cross-sectional study, we found that 11.6% of PLWH in the city of Bucaramanga (Colombia) were co-infected with HBV when the nucleic acid testing was performed on all specimens, regardless of HBV serologic profile. The local prevalence in the northeastern region of Colombia was higher than previously reported in the country (between 2.9 and 3.3%), probably because only the HBsAg test was included in the serological screening (Tengan et al., 2017). Nevertheless, the high frequency of HIV/HBV co-infection reported in the present study was similar to that reported in Latin American countries, such as Brazil and Cuba with10.3% (Corredor et al., 2005; Soares Sampaio et al., 2009) or in the Caribbean with 13.55% in Haiti (Schweitzer et al., 2015). The regional prevalence of HBV in the Americas may have increased in the last five years, as the Pan American Health Organization estimates that the annual infections with the HIV virus rose by 21% from 2010 through 2021 (Pan American Health Organization, 2021).

We found no statistically significant differences in HIV viral load and CD4+ cell counts between HIV/HBV co-infected and mono-infected patients. This observation has been replicated in previous studies (Wondimeneh et al., 2013; Sarkar et al., 2016) but other authors have demonstrated lower baseline CD4+ cell counts, increased HIV viral load, and AIDS-related comorbidities in HIV-HBV co-infected patients [Idoko et al., 2009; Thio et al., 2013; The Canadian Observational Cohort (CANOC) Collaboration et al., 2019]. Lower CD4 counts have been associated with higher levels of HBV viral replication (HBV DNA > 200,000 IU/mL) and may increase the risk of developing HCC (Clifford et al., 2008). In Colombia, hepatopathies are a major cause of morbidity and mortality among PLWH. Future longitudinal studies in a large cohort will allow investigation of the etiology and progression of chronic liver disease.

Here, we reported genotype F3 in all co-infected patients. These results are limited to Colombia, where our group and others have described that genotype F3 was predominant (>75%) in different populations such as blood donors, PLWH, patients with cirrhosis and HCC, followed in a lesser proportion by genotypes F1b, A, and G (Alvarado Mora et al., 2011; Bautista-Amorocho et al., 2014; Duque-Jaramillo et al., 2017). The results also showed that most PLWH were receiving HAART (89.3%), and of these, 56.7% were receiving a combination of NRTI + NNRTI, followed by NRTI + PI (22.8%). In both combinations, 96.7% had LAM as an NRTI drug and none had TDF.

To the best of our knowledge, this is the first study in Colombia to investigate antiviral resistance mutation profiles of the HBV *pol* gene associated with NRTI treatment. Of the 12 known codons that can confer drug resistance along the conserved RT domain of HBV, seven were identified in the present study. All HBV sequences (*n* = 23) from PLWH were on HAART and showed mutations conferring clinical resistance to different NRTIs. The most common were the triple amino acid substitutions rt80I, rt180M, and rt204V, which are associated with LAM and LdT resistance and were found in 91.3% of the cases. Interestingly, we also observed the triple combination of antiviral resistance rt80I, rt180M, and rt204A, described as compensatory or secondary mutations in patients undergoing ETV therapy. This pattern of amino acid substitutions can restore RT activity, as a consequence of defects caused by primary resistance (Delaney et al., 2003; Deng and Tang, 2011; Lee et al., 2012). Thus, the replication potential of HBV could persist due to compensatory mutation. A high prevalence of LAM resistance mutations has also been reported in previous studies in South Africa in treatment-naïve and treatment-experienced individuals with CHB and OBI mostly infected with genotype A1 (Selabe et al., 2007). In France, 90% of rt180M and rt204V LAM resistance patterns were estimated in PLWH after 48 months of treatment (Benhamou et al., 1999). Similarly, in an international multi-cohort study, three different resistance mutation patterns (rt204V/I, rt180M plus rt204V/I, and rt173L plus rt180M plus rt204V/I) were observed in 50 and 94% of patients after 2 years and 4 years of LAM-based HAART, respectively (Matthews et al., 2006). Finally, in India, double (rt180M, rt204V) and triple (rt173L, rt180M, rt204V) mutations associated with genotype D were found in HIV/HBV coinfected patients after 24–48 months of antiviral therapy (Pal et al., 2015). In Latin America, there are no previous reports of RAMs to LAM or other NRTIs in HIV/HBV co-infected patients on HAART.

Due to the organization of the HBV genome, the *S* gene which encodes the envelope proteins (small, medium and large), is completely overlapped by the *pol* gene. Therefore, mutations in the *pol* ORF associated with antiviral RAMs, may result in changes in the HBsAg that interfere with the vaccine-derived protection (Torresi, 2002). In patients where LAM has been widely used continuously for several years, scape mutants and/or ADAPVEMs with nucleotide variation within the *S* gene are likely to occur. The current results show ADAPVEMs in all HIV/HBV co-infections associated with LAM and LdT treatment. However, no vaccine escape mutants were observed. In the present study HBV vaccine coverage was relatively low (17.1%); therefore, NRTI treatment as part of the HIV HAART, has the potential to cause the emergence of ADAPTVEMs and probably spread escape mutants in HBV-infected individuals.

Our study had several limitations. First, HBV viral load and alanine aminotransferase (ALT) were not measured due to insufficient sample volumes. Second, adherence to HAART or duration of treatment could not be confirmed in mono-and co-infected PLWH. Third, a cohort of HIV/HBV antiretroviral treatment-naïve and CHB mono-infected individuals should be included in future studies to characterize natural drug resistance mutations associated with genotype F circulation in Colombia.

## 5. Conclusion

The prevalence of HIV/HBV coinfection in Colombian patients was high, mainly associated with OBI cases. Therefore, nucleic acid amplification testing is recommended for HBV screening regardless of HBsAg, anti-HBs and anti-HBc serologic status. Most NRTI-resistant mutant strains correspond to low genetic barrier antivirals such as Lamivudine an Telbivudine. The American Association for the Study of Liver Diseases (AASLD) discourages the use of LAM as the sole antiviral drug to treat HBV infection within the HIV HAART regimen, due to its low potency and the high risk of mutations that confer cross-resistance to other anti-HBV drugs such as Emtricitabine, Telbivudine, and Entecavir (Terrault et al., 2016). Therefore, a high genetic barrier HBV antiviral such as Tenofovir Disoproxil Fumarate or Tenofovir Alafenamide plus Lamivudine is recommended as first-line of HAART in HIV co-infection (Thio et al., 2015; Wandera et al., 2022).

## Data availability statement

The datasets presented in this study were uploaded in online repositories. The names of the repository/repositories and accession number(s) can be found at: https://www.ncbi.nlm.nih.gov/genbank/, OQ262971-OQ262995.

## Ethics statement

The studies involving human participants were reviewed and approved by Ethics Committee of the Universidad de Santander. The patients/participants provided their written informed consent to participate in this study.

## Author contributions

HB-A designed the study, recruited the patients, performed the sequencing, bioinformatics analysis, and drafted the manuscript. HB-A, JP-V, and JAS-S performed the experiments and analyzed the data. JAS-S prepared the tables, performed the statistics, and revised the manuscript. JP-V designed the figures. All authors have read and approved the final version of the submitted manuscript.

## Conflict of interest

The authors declare that the research was conducted in the absence of any commercial or financial relationships that could be construed as a potential conflict of interest.

## Publisher’s note

All claims expressed in this article are solely those of the authors and do not necessarily represent those of their affiliated organizations, or those of the publisher, the editors and the reviewers. Any product that may be evaluated in this article, or claim that may be made by its manufacturer, is not guaranteed or endorsed by the publisher.

## Funding

This study was funded by Ministerio de Ciencia y Tecnología (MinCiencias) of Colombia, Grant 758-2018 code number 129977757637 and Vicerrectoría de Investigaciones de la Universidad de Santander (UDES).

## Footnotes

^1^ https://hbv.geno2pheno.org/
